# Dry Matter Production, Photosynthesis of Flag Leaves and Water Use in Winter Wheat Are Affected by Supplemental Irrigation in the Huang-Huai-Hai Plain of China

**DOI:** 10.1371/journal.pone.0137274

**Published:** 2015-09-03

**Authors:** Jianguo Man, Yu Shi, Zhenwen Yu, Yongli Zhang

**Affiliations:** Key Laboratory of Crop Ecophysiology and Farming System, Ministry of Agriculture, College of Agronomy, Shandong Agricultural University, Taian, Shandong, 271018, P.R. China; Universidade Federal de Viçosa, BRAZIL

## Abstract

Winter wheat is threatened by drought in the Huang-Huai-Hai Plain of China, thus, effective water-saving irrigation practices are urgently required to maintain its high winter wheat production. This study was conducted from 2012 to 2014 to determine how supplemental irrigation (SI) affected soil moisture, photosynthesis, and dry matter (DM) production of winter wheat by measuring the moisture in 0–20 cm (W2), 0–40 cm (W3), and 0–60 cm (W4) soil profiles. Rainfed (W0) and local SI practice (W1, irrigation with 60 mm each at jointing and anthesis) treatments were designed as controls. The irrigation amount for W3 was significantly lower than that for W1 and W4 but higher than that for W2. The soil relative water content (SRWC) in 0–40 cm soil profiles at jointing after SI for W3 was significantly lower than that for W1 and W4 but higher than that for W2. W3 exhibited lower SRWC in 100–140 and 60–140 cm soil profiles at anthesis after SI and at maturity, respectively, but higher root length density in 60–100 cm soil profiles than W1, W2 and W4. Compared with W1, W2 and W4, photosynthetic and transpiration rates and stomatal conductance of flag leaves for W3 were significantly greater during grain filling, particularly at the mid and later stages. The total DM at maturity, DM in grain and leaves, post-anthesis DM accumulation and its contribution to grain and grain filling duration were higher for W3. The 1000-grain weight, grain yield and water use efficiency for W3 were the highest. Therefore, treatment of increasing SRWC in the 0–40 cm soil profiles to 65% and 70% field capacities at jointing and anthesis (W3), respectively, created a suitable soil moisture environment for winter wheat production, which could be considered as a high yield and water-saving treatment in Huang-Huai-Hai Plain, China.

## Introduction

The Huang-Huai-Hai Plain is one of the most important agricultural regions in China and produces more than 60% of China’s winter wheat [1]. This area has a warm, temperate, semi-humid continental monsoon climate. The average yield of winter wheat is approximately 5540 kg ha^−1^, and yields in excess of 9000 kg ha^−1^ have been reported by farmers in the region. However, water deficits have threatened the winter wheat production in this region. The mean annual precipitation in this region is approximately 500–600 mm; however, approximately only 30%–40% of the rain occurs in winter wheat growing seasons [2–4]. The total water consumption required by high-yield winter wheat is 400–500 mm during the growing season [3, 5]. Therefore, additional irrigation is required to maintain a high winter wheat production. The traditional practice in this region is to irrigate winter wheat with up to 310 mm water, which lies within the crop evapotranspiration (*ETc*) range for the maximum water use efficiency (WUE) and maximum yield; however, the WUE is only 13.2 kg ha^−1^ mm^−1^ [6, 7], which is lower than that of most wheat producing regions in the world (17.0 kg ha^−1^ mm^−1^ on average) [8]. Therefore, effective water-saving technologies are urgently needed, particularly those that seek to match crop water demand with SI by considering precipitation, soil water storage and *ETc* [9].

Supplemental irrigation (SI) is a highly efficient practice with the potential to save irrigation water, increase agricultural production and improve livelihoods. Despite several water-saving practices, e.g. Gao et al. [10], Zhang et al. [11], Dong et al. [12], Fang et al. [13], Li et al. [14], and Lv et al. [15], have been proposed in the last years, most of them focused on fixed amounts of irrigation and, nowadays, application of 60 mm each at jointing and heading is the more commonly SI used in the Huang-Huai-Hai Plain [16]. However, there are some limitations in these studies, as they did not consider the effect of soil water conditions before irrigation (particularly those at different soil depths) on the irrigation amount, water consumption and winter wheat production.

The grain yield of a crop is dependent on the dry matter (DM) accumulation by photosynthesis of leaves post-anthesis and DM remobilization (DMR) [17, 18], which are closely related to irrigation and soil moisture conditions. Leaf photosynthetic capacity is a key parameter determining crop yield; it is enhanced by moderate soil moisture and reduced in both severe water deficit and excessive water conditions [19]. Our previous study by Guo et al. [20] determined the irrigation amount by measuring the soil moisture at soil depths of 0–20 cm (D20), 0–40 cm (D40), and 0–60 cm (D60) to investigate the response of photosynthetic characteristics of flag leaves to the SI. The results showed that the total irrigation in the D40 treatment were 62.4 mm and 118.2 mm in 2011–2012 and 2012–2013 growing seasons, respectively, which were 15.3 mm and 54.0 mm higher than D20, but 23.1 mm and 21.0 mm lower than D60 in the two growing seasons, respectively. The photosynthetic rate (*P*_n_), transpiration rate (*T*_r_) and stomatal conductance (*g*_s_) of flag leaves in D40 were significantly higher than those in D20 and D60; however, the photosynthesis observations were limited to the early grain-filling stage [14 days after anthesis (DAA)], which may not represent the photosynthetic characteristics during all the grain-filling stages, especially at the later stages.

Increasing DM accumulation (DMA) at maturity and improving the contribution of post-anthesis DM to grain can improve grain filling and thus increase grain yield [17, 21, 22, 23, 24]. The previous study by Guo et al. [20] showed that the grain-filling rate (GFR) of D40 was significantly higher than that of D20 and D60 from 14 DAA to 28 DAA, and the highest grain yield was obtained in D40. However, the performance of the DMA at anthesis and maturity, DMR post-anthesis and the characteristics of grain filling [i.e. the duration of grain filling (DGF) and the average rate] for the D40 treatment are relatively limited.

In the present study, we recharged the soil moisture to 65% field capacity (FC) at jointing and 70% FC at anthesis in three soil profiles of 0–20 cm, 0–40 cm and 0–60 cm to determine the irrigation amount as described in our previous study by Guo et al. [20] during the winter wheat growing seasons from 2012 to 2014; a rainfed treatment and a local SI practice treatment (60 mm of irrigation each at jointing and anthesis) were designed as controls for the following aims: (1) to make further efforts to study the effect of SI on the soil water condition and root distribution at anthesis, (2) to determine the photosynthetic characteristics of flag leaves during all the grain-filling stages and (3) to investigate DMA post-anthesis, remobilisation and the characteristics of winter wheat grain filling.

## Materials and Methods

### Ethics Statement

The research station of this study is a department of Shandong Agricultural University. The farming operations of this experiment were similar to the rural farmers’ operations and did not involve endangered or protected species; the operations were approved by the Key Laboratory of Crop Ecophysiology and Farming System of Ministry of Agriculture, Shandong Agricultural University.

### Experimental site

**Fig 1:**
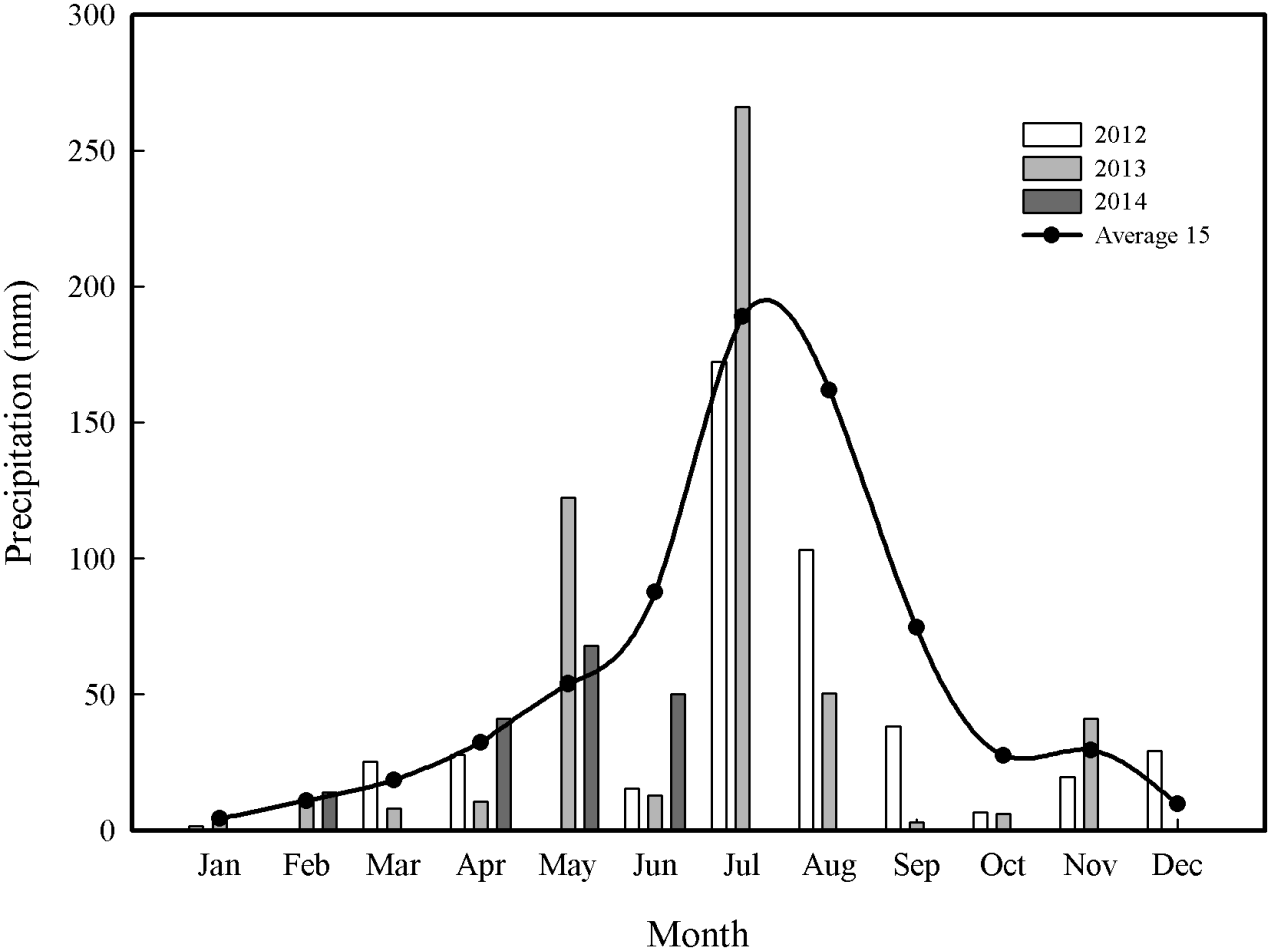
The monthly precipitation in 2012 to 2014 and the average value during the recent 15 years (Average 15). Precipitations amounts from July to December were not measured in 2014.

Field experiments were conducted from October 2012 to June 2014 in Shijiawangzi Village, Yanzhou, Shandong Province, China (116°41′E, 35°42′N). This village is located in the center of the Huang-Huai-Hai Plain, and its environment is typical and representative of the plain. The area has a warm temperate semi-humid continental monsoon climate and an annual average temperature of 13.6°C. The area has 2461 h of annual accumulated sunshine and an annual precipitation of 621.2 mm (228.8 mm during the winter wheat growing season). The groundwater depth is 25 m. The organic matter, total nitrogen, available phosphorus, and available potassium in the topsoil (0–20 cm) of the experimental plots were 15.9 g kg^−1^, 1.2 g kg^−1^, 30.9 mg kg^−1^, and 114.5 mg kg^−1^, respectively. The soil bulk density and soil water of the top 0–200 cm of the soil (in 20-cm increments) are shown in Table 1. The amounts of precipitation during the growing seasons in 2012/2013 and 2013/2014 were 225.0 and 170.0 mm, respectively, and the monthly precipitation from 2012 to 2014 and the average value during the most recent 15 years are shown in Fig 1.

**Table 1:** Soil bulk density and soil water content of 20 cm layers in the top 0–200 cm soil profile of the experimental field.

Soil profiles (cm)	Soil bulk density (g cm^-3^)		Field capacity (mg water g^−1^ dry soil)		Soil relative water content one day before sowing (%)	
	2012–2013	2013–2014	2012–2013	2013–2014	2012–2013	2013–2014
0–20	1.4	1.4	286.7	290.0	60.9	68.2
20–40	1.4	1.4	261.7	280.0	61.6	68.7
40–60	1.5	1.4	270.1	270.0	52.9	75.0
60–80	1.5	1.5	260.3	260.0	62.8	70.8
80–100	1.5	1.5	254.1	260.0	73.8	74.1
100–120	1.5	1.5	259.1	252.8	75.3	80.8
120–140	1.5	1.5	251.8	249.7	85.0	80.6
140–160	1.5	1.5	248.1	245.7	87.9	84.6
160–180	1.6	1.6	232.8	241.4	92.9	85.7
180–200	1.6	1.5	238.4	240.3	91.9	88.2

### Experimental design and irrigation management

The following five treatments were designed: a rainfed (W0) treatment with no irrigation; a local SI practice treatment (W1, 60 mm of irrigation at both jointing and anthesis); and three treatments in which the soil profiles were measured at ranges of 0–20 cm (W2), 0–40 cm (W3) and 0–60 cm (W4) for soil water content (SWC) prior to SI. SI brought the SWC in each measured soil profile to 65% FC at jointing (Z31, first node detectable) and 70% FC at anthesis (Z61, beginning of anthesis) [25]. Before irrigation, SWC was measured in the corresponding soil layers to calculate the appropriate amounts of SI using the equation described by Guo et al. [20] and Jalilian et al. [26]:

$$CIR=100y_{bd}D_{h}\left(\theta_{t}-\theta_{n}\right),$$

where *CIR* (mm) is the amount of SI, *y*_*bd*_ (g cm^−3^) is the soil bulk density, *D*_*h*_ (cm) is the thickness of the soil profile measured for SWC before irrigation, *θ*_*t*_ (mg water g^−1^ dry soil) is the target SWC on a weight basis after SI, and *θ*_*n*_ (mg water g^−1^ dry soil) is the water content of the soil on a weight basis before irrigation. *θ*_*t*_ was calculated as follows:

$$\theta_{t}=\theta_{\text{max}}\theta_{ty}/100,$$

where *θ*_max_ (mg water g^-1^ dry soil) is the field capacity and *θ*_*tr*_ (%) is the target relative SWC. Water was sprayed though plastic hoses evenly onto the experimental plots under pressure. A flow meter was used to measure the amount of water applied. CIR for different treatments is shown in Table 2.

**Table 2:** The target soil relative water content (θ_t_) and actual soil relative water content (θ_a_) in the supplemental irrigation (SI) treatments (W2, W3 and W4) after jointing and anthesis in 2012–2013 and 2013–2014 growing season, the amount of supplemental irrigation (CIR) is also indicated.

Treatment ^a^	Thickness of soil profile (cm)	SI at jointing			SI at anthesis			Total CIR (mm)
		θ_t_(%)	θ_a_(%)	CIR(mm)	θ_t_(%)	θ_a_(%)	CIR(mm)	
2012–2013								
W2	20	65.0	64.7	30.7	70	71.1	32.0	62.8
W3	40	65.0	63.5	49.6	70	68.2	51.2	100.8
W4	60	65.0	63.5	65.8	70	67.9	66.7	132.5
2013–2014								
W2	20	65.0	64.1	23.8	70	68.2	24.5	48.3
W3	40	65.0	63.5	44.0	70	70.3	45.5	89.6
W4	60	65.0	63.5	58.0	70	72.8	66.7	124.7

^a^ The treatment of W2, W3 and W4 were supplemental irrigation determined by measuring 0–20 cm, 0–40 cm and 0–60 cm soil profile moisture, respectively, and brought the soil moisture to 65% field capacity (FC) at jointing and 70% FC at anthesis.

### Crop management

All plots were supplied with 240 kg N ha^-1^, 150 kg P_2_O_5_ ha^-1^ and 150 kg K_2_O ha^-1^. All P and K fertiliser and half the N fertiliser were applied pre-sowing. The remaining N fertiliser was top-dressed at the jointing stage. The high-yielding wheat (*Triticum aestivum* L.) cultivar Jimai22 was used in the experiments. The wheat seeds were sown at a density of 180 plants m^-2^ on October 10, 2012, and October 9, 2013. Wheat seedling shoots ceased growth at the beginning of December and started to grow again at the end of February. During this period, the average daily temperature was below 0°C. Wheat plants were harvested on June 12, 2013 and June 6, 2014.

### Crop water use

The soil samples were collected with a soil corer in 20-cm increments to a depth of 200 cm in all experimental plots. SWC (gravimetric water content, mg water g^−1^ dry soil) was determined using the oven-drying method [27]. The measurements were performed before sowing (Z00) and 1 day before irrigation and 3 days after irrigation at jointing (Z31), at anthesis (Z61), and at maturity (Z90). Three soil samples were taken at random locations from each plot.

The total water consumption or *ETc* was calculated using the soil water balance equation [28] for the growing season:

$$ET_{c}=P+CIR+\Delta W-R-D,$$

where *ET*_*c*_ (mm) is the total water consumption during a growing season, *P* (mm) is the precipitation, *CIR* (mm) is the amount of SI, Δ*W* (mm) is the soil water storage at sowing minus the soil water storage at harvesting for the 0–200 cm soil profile, *R* (mm) is the surface runoff, and *D* (mm) is the downward flux below the crop root zone. The soil water measurements indicated that the drainage at the site is negligible; therefore, deep percolation was not accounted for in the present study [15, 29].

### Root sampling

Roots sampled from three replicates at each plot, according to the description of Xue et al. [30] and Zhang et al. [31], were taken at anthesis. Prior to root sampling, the aboveground parts were carefully removed, and the samples were collected from soil cores (10.0 cm diameter) with 20-cm increments to 100 cm at anthesis [31]. Two cores per replicate in a plot were collected: one within the crop row and one midway between rows [30]. The resultant mixture of roots and organic debris was then placed in a polythene bag and washed with tap water. The mixture was carefully separated and then preserved by refrigeration for further testing. Before measuring the cleaned roots, we stained them with methyl blue for at least 12 h at 4°C. Roots were arranged and floated on shallow water in a transparent tray (0.24 × 0.32 m), scanned with a flatbed scanner (HP Scanjet 8200; Hewlett–Packard, Palo Alto, CA, USA) at a resolution of 600 dpi, and then analyzed using an image analyzer (Delta-T Area Meter Type AMB2; Delta–T Devices Ltd., Cambridge, UK) to measure root lengths [32]. The root length density (RLD; cm cm^−3^) was calculated by dividing the total root lengths by the volume of the corresponding soil core section.

### Photosynthetic rate, transpiration rate, and stomatal conductance

The flag leaf *P*_n_, *T*_r_ and *g*_s_ were measured using a CIRAS-2 Portable Photosynthesis System (PP-Systems, Hitchin, UK) on ten flag leaves under natural conditions with 380–420 μmol CO_2_ mol^-1^ air, 1100–1400 μmol m^−2^ s^−1^ photosynthetically active radiation (PAR) and 28–30°C from each experimental plot. All measurements were made between 9:00 and 11:00 on days with full sunlight at 7-day intervals from anthesis to 35 DAA.

### DM production and grain filling rate

Plant samples were collected to determine DM on the following five occasions: before winter (Z25), during regreening (Z26), when the first node was detectable (Z31), at the beginning of anthesis (Z61), and at maturity (Z90). On each occasion, 20 consecutive plants were manually cut at ground level from each experimental plot. These plants were separated into leaves, stems plus sheaths, and spikes at anthesis; the plants were separated into leaves, stems plus sheaths, grains, and spike axes plus glumes at maturity. All samples were dried to a constant weight in a forced-draught oven at 70°C, and their dry weight was then recorded. The plant density for each plot was determined as the mean value of one square meter.

The following parameters related to DM accumulation and remobilization within the wheat plant were calculated following Arduini et al. [33] and Masoni et al. [34]:

DM accumulation post-anthesis (DMA_p_) as the difference between DM content of the whole plant at anthesis and at maturity.

$$\begin{array}{l} \text{DM remobilization }\left(\text{DMR}\right)=\text{DM of the aerial plant part at anthesis }-\;(\text{DM of stem plus sheaths},\;\\ \text{leaves},\text{ spikes axis plus glumes at maturity}) \end{array}$$

The contribution of DMA to grain (CDMAG, %)=DMA / DM of grain at maturity ×100

The contribution of DMR to grain (CDMRG, %)=DMR / DM of grain at maturity ×100

### Grain filling traits

Emerging flowering spikes were all tagged on the same day. Twenty tagged spikes from each experimental plot were sampled at 7-day intervals from the beginning of anthesis (Z61) to maturity (Z90). The grain-filling rate was estimated from the accumulation of grain dry weight. At each sampling date, grains were separated from the glumes and dried at 105°C for 10 min and then at 70°C until reaching a constant weight. The total number of grains was determined, and their dry weight was recorded.

Grain filling can be modeled using a logistic function [35, 36]:

GW=K/(1+exp(A−Bt)),

where *GW* (g) is the 1000-grain weight during grain filling, *K* (g) is the upper asymptote of 1000-grain weight, *A* and *B* are constants that determine the curvature of the relationship, and *t* (days) is the grain filling time. The duration of grain filling (*T*; days) and the mean value of grain filling rate (Va, mg grain^−1^ day^−1^) can be obtained from the logistic function and its first and second derivatives. *T* and *V*_*a*_ were calculated as follows:

T=(4.59512+A)/B

and

$$V_{a}=K/T.$$

### Grain yield and WUE

The grain yield was determined from each experimental plot and is reported on the basis of 12.5% moisture content basis.

The WUE of winter wheat was calculated as follows [37]:

$$WUE=Y/ET_{c},$$

where *WUE* (kg ha^−1^ mm^−1^) is the WUE for grain yield, *Y* (kg ha^−1^) is the grain yield and *ET*_*c*_ (mm) is the total ETc over the winter wheat growing season.

### Statistical analysis

The data were subjected to an ANOVA (SSPS for Windows, version 13.0). The ANOVA used a level of significance of α = 0.05 to identify significant differences between treatments. For determining significant effects between treatments, multiple comparisons were made using the least significant difference test with α = 0.05.

## Results

### Soil moisture conditions after irrigation

**Fig 2:**
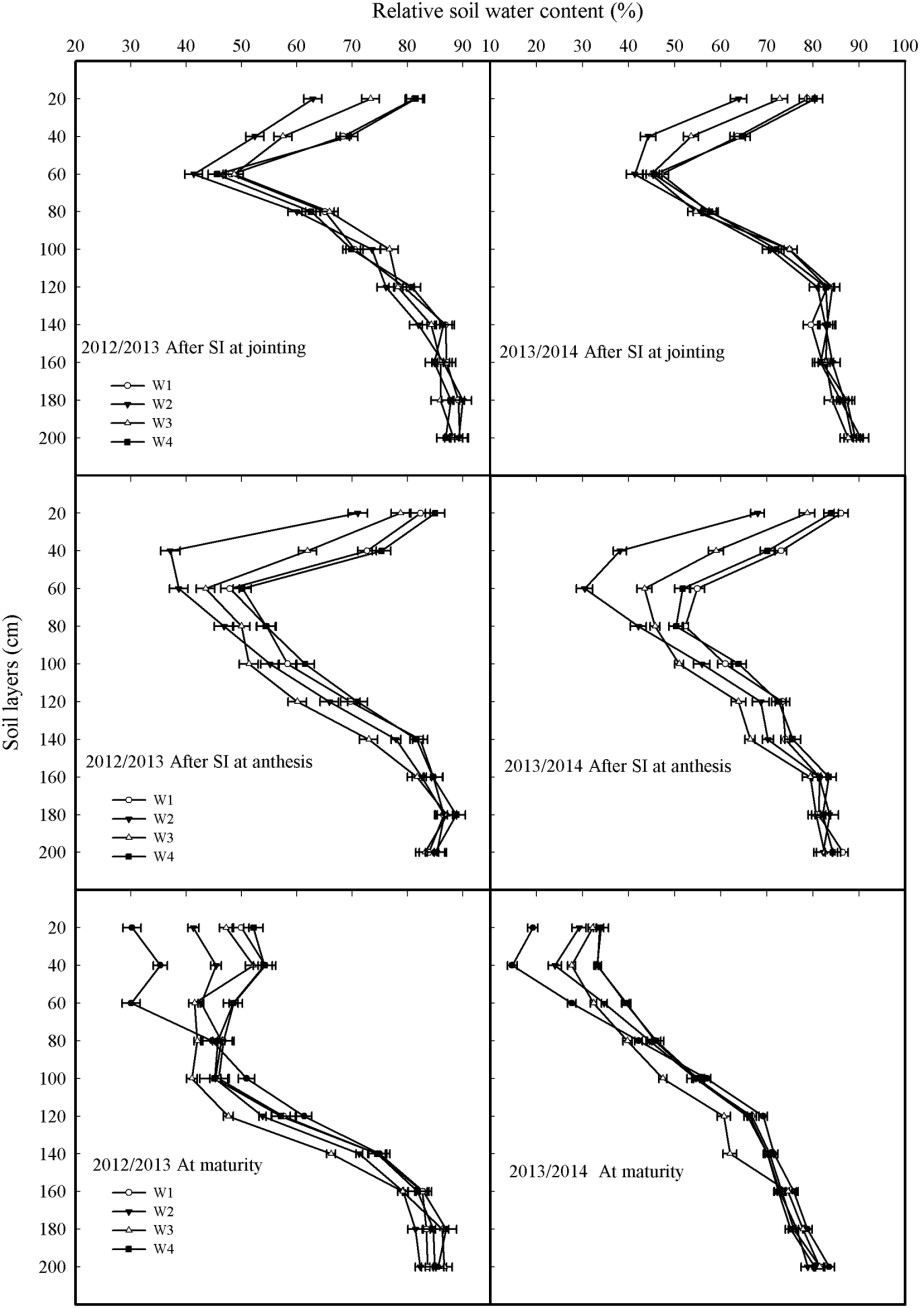
Soil relative water content in 0–200 cm soil profiles after irrigation and at maturity of different treatments in 2012–2013 and 2013–2014 growing seasons. Rainfed (W0), a local SI with 60 mm each at jointing and anthesis (W1), SI by measuring 0–20 cm (W2), 0–40 cm (W3), and 0–60 cm (W4) soil moisture and brought the soil moisture to 65% field capacity (FC) at jointing and 70% FC at anthesis. Error bars represent standard errors of the means (P<0.05).

Fig 2 shows SRWC after SI at jointing and anthesis in both the 2012–2013 and 2013–2014 growing seasons. At jointing, SRWCs of the 0–40 cm soil profiles from the W1 and W4 treatments did not differ and were significantly higher than those from the W3 treatment. The lowest SRWC was from W2. SRWCs of the 60–200 cm soil profiles among the treatments did not differ. At anthesis, SRWC of the 0–140 cm soil profiles from W1 and W4 treatments was significantly higher than that from either W2 or W3. Furthermore, compared with W2, W3 had a higher SRWC of 0–80 cm soil profiles but lower SRWC of 100–140 cm soil profiles. SRWCs of the 160–200 cm soil profiles in the treatments were not different in 2012–2013 and 2013–2014.

SRWCs at maturity are also shown in Fig 2. In 2012–2013, SRWCs of the 0–140 cm soil profiles from the W1 and W4 treatments did not differ and were significantly higher than those from W2 and W3. Compared with W2, W3 had a higher SRWC of 0–40 cm soil profiles but lower SRWC of 60–140 cm soil profiles. In 2013–2014, there were no differences in SRWCs of the 0–20 cm soil profile between W1 and W3 or W4; however, all were significantly higher than those from W2. SRWCs of the 20–80 cm soil profiles from W1 and W4 were significantly higher than those from W2 and W3. SRWCs of the 80–140 cm soil profiles from W1 and W2 or W4 were not different but were significantly higher than those from W3. These results indicate that SI provided in the W3 treatment promoted water consumption in the 60–140 cm soil profiles.

### Root length density

**Fig 3:**
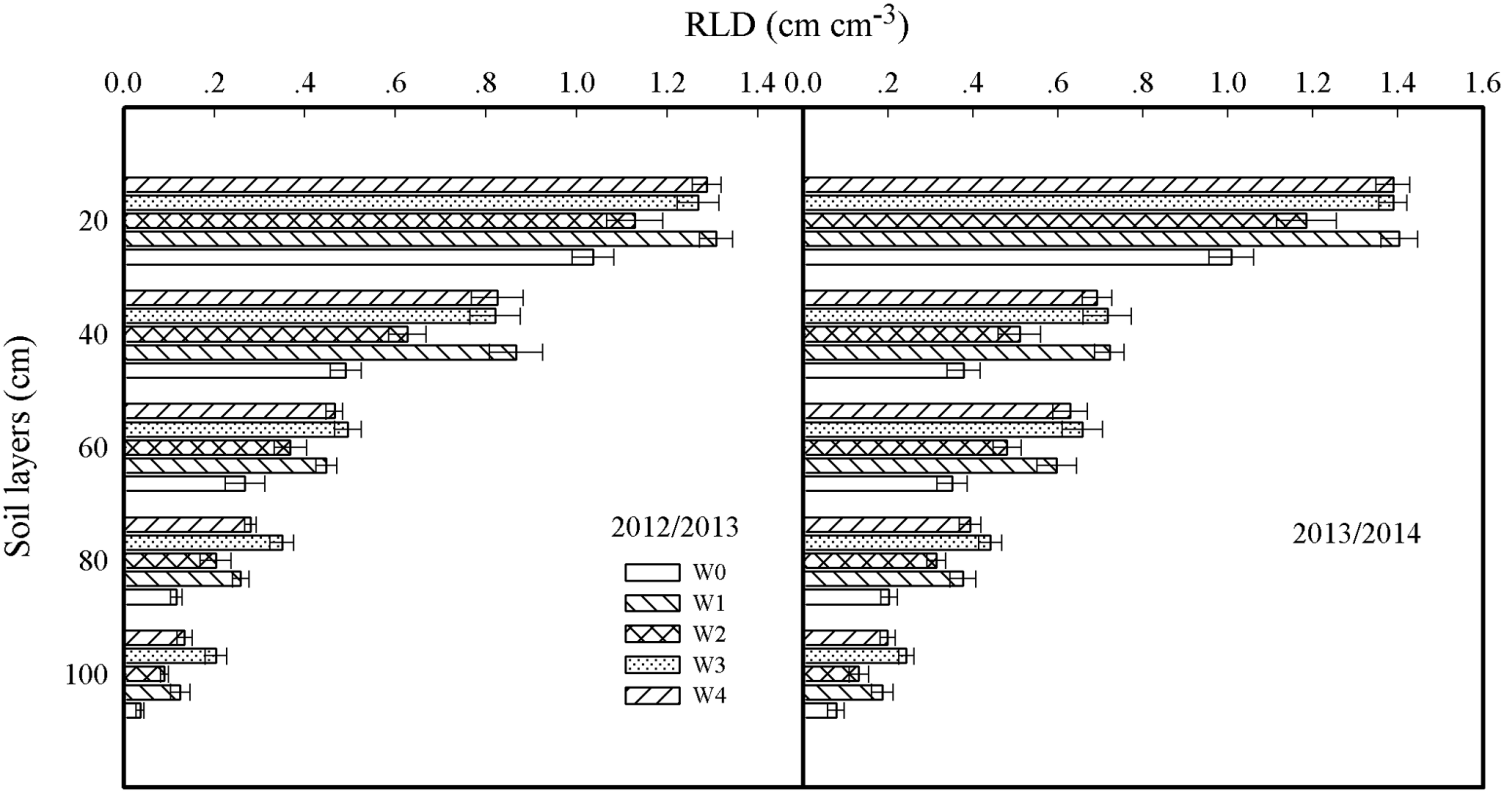
Root length density (RLD) at anthesis in 2012–2013 and 2013–2014 growing seasons. Error bars represent standard errors of the means (P<0.05).

The root length density (RLD) of the 0–100 cm soil layers at anthesis is presented in Fig 3. RLD data measured in 2013–2014 exhibited similar differences between treatments to those measured in 2012–2013. The mean values of RLD in the 0–100 cm soil profiles from W3 were the highest at 0.63 and 0.69 cm cm^−3^ in the 2012–2013 and 2013–2014 growing seasons, respectively. In the 0–40 cm soil layers, RLD from W3 exhibited no differences compared with those from W1 and W4. In the 40–100 cm soil layers, W3 demonstrated a higher RLD than W1 and W4, and the differences were significant for both the 60–80 cm and 80–100 cm soil layers, which is likely the reason for the higher water consumption in the soil layers under 60 cm in W3 than for W1 and W4. RLD in the 0–100 cm soil layers from W2 was significantly lower than that from W1, W3 and W4 but higher than that from W0.

### Photosynthetic characteristics

**Fig 4:**
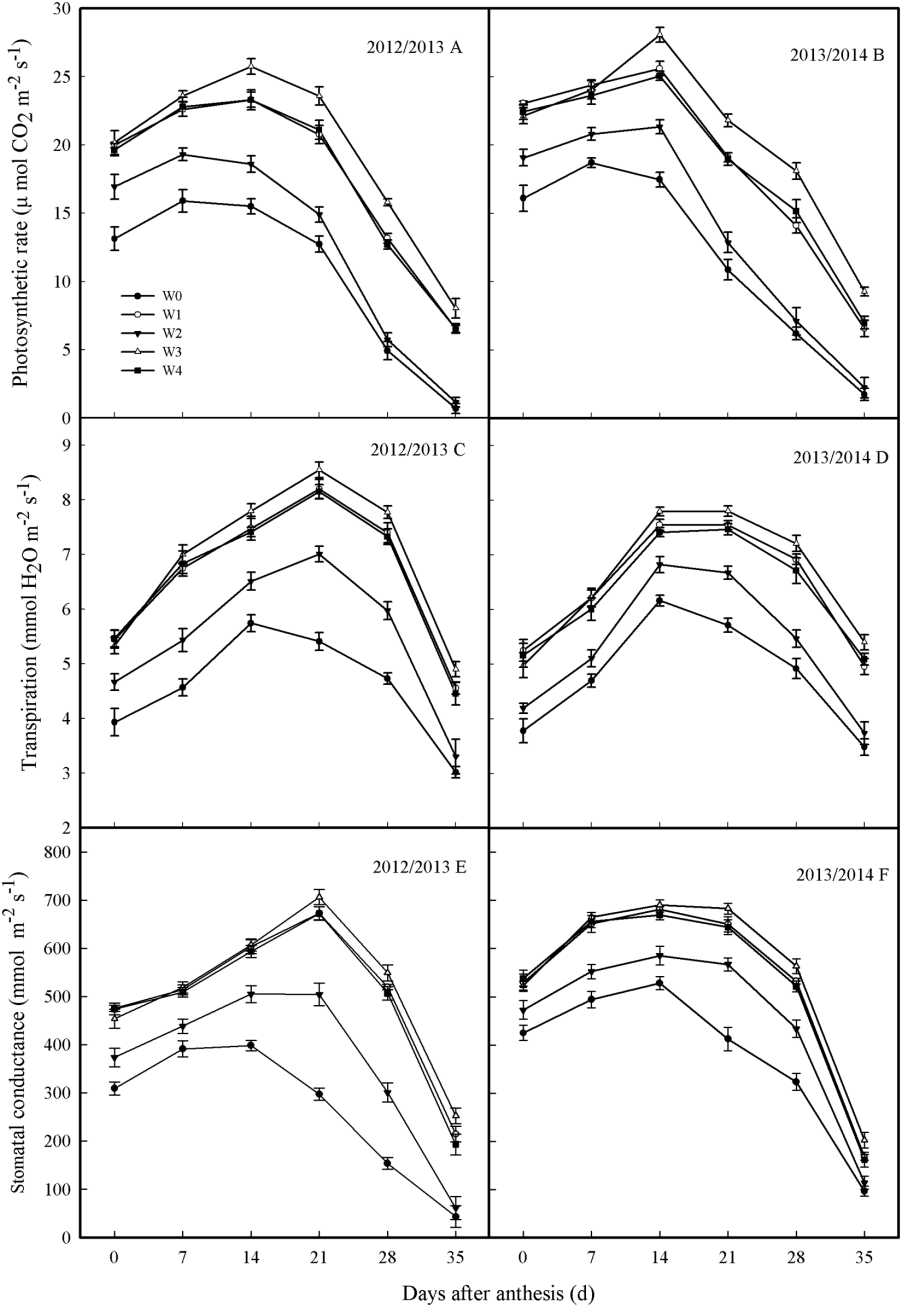
Photosynthetic rate (*P*_n_, A and B), transpiration rate (*T*_r_, C and D) and stomatal conductance (*g*_s_, E and F) during grain filling in 2012–2013 and 2013–2014 growing seasons. Error bars represent standard errors of the means (P<0.05).

There were no differences in the *P*_n_, *T*_r_ or *g*_s_ of plants between the W1, W3 and W4 from anthesis to 7 DAA (Fig 4). The *P*_n_ of plants from the W3 was significantly higher than those of plants from the W1 and W4 at 14 DAA; *T*_r_ and *g*_s_ exhibited no differences among these treatments at this time point. From 21 to 35 DAA, the *P*_n_, *T*_r_ and *g*_s_ of plants from the W3 were significantly higher than those of plants from the W1 and W4; however, these values did not differ between the W1 and W4. W2 exhibited the lowest values of *P*_n_, *T*_r_ and *g*_s_ among the SI treatments. This pattern indicates that SI determined by measuring 0–40 cm soil moisture could improve the photosynthesis capacity during grain filling stages, particularly at mid and later stages, which is conducive to the increase in grain weight.

### Dry matter accumulation at anthesis and maturity

**Fig 5:**
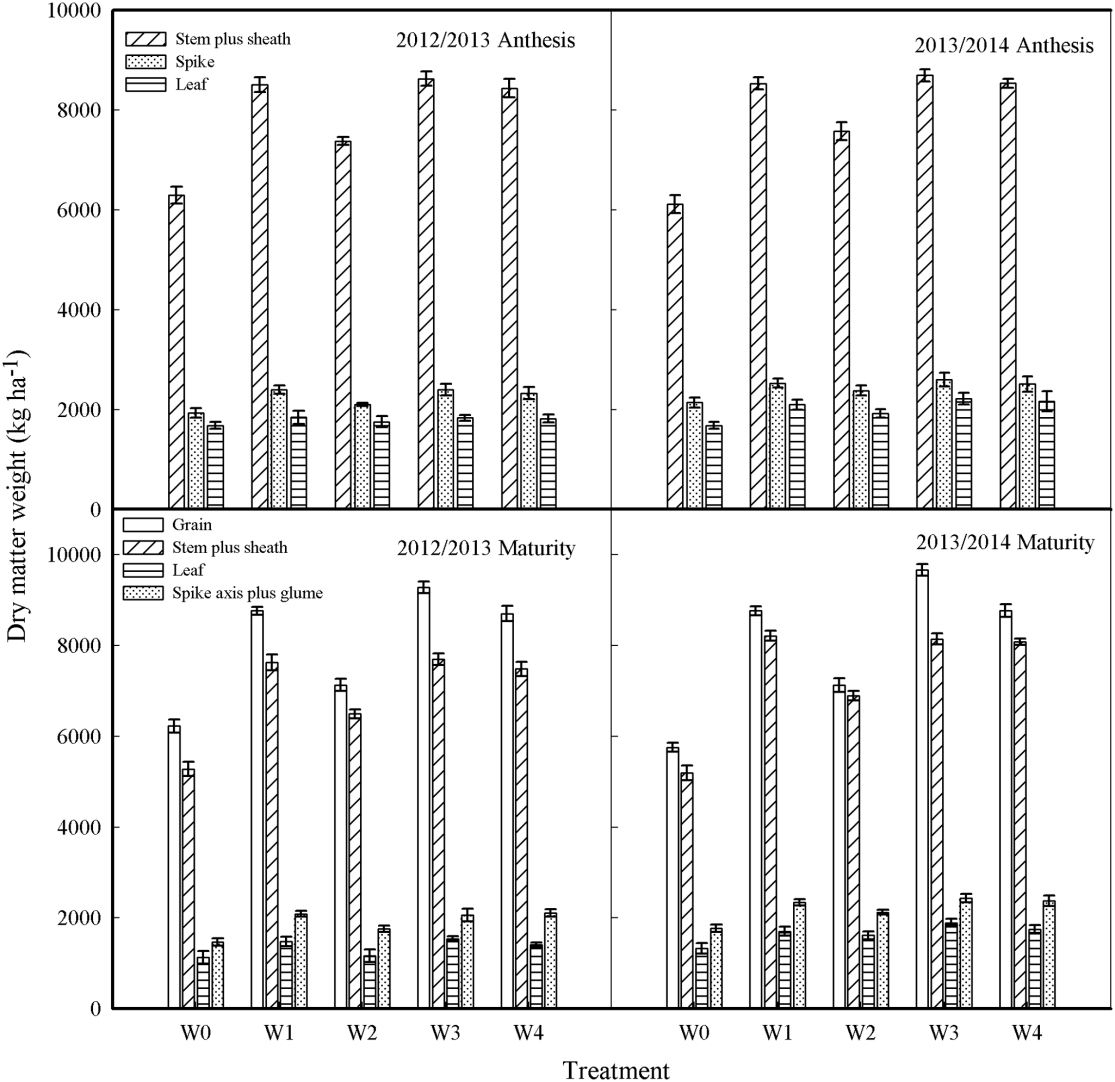
Dry matter accumulation in different organs of the plant at anthesis and maturity from different treatments in 2012–2013 and 2013–2014 growing seasons. Error bars represent standard errors of the means (P<0.05).

The total DMA and DM in each organ at anthesis from the W1, W3, and W4 treatments was not different but significantly greater than that from the W0 and W2 treatments in both growing seasons (Fig 5). DM in leaves at anthesis among the treatments did not differ in 2012–2013. However, DM from the W1, W3, and W4 treatments was significantly higher than that from the W0 and W2 treatments in 2013–2014.

At maturity, DM in grain and leaves from the W3 treatment was the greatest. The next highest values were from the W1 and W4 treatments. The lowest values were from the W2 treatment (Fig 4). DM values in stems plus sheaths and in spike axes plus glumes from the W1, W3, and W4 treatments did not differ but were significantly higher than those from the W2 treatment. DM values in grain, stems plus sheaths, leaves, and spike axis plus glumes from the W0 treatment were the lowest in both growing seasons.

### Contribution of DMR and DMA post-anthesis to grain

DMR and its contribution to grain in W1, W3 and W4 were lower than those in the W2. However, DMA post-anthesis (DMA_p_) and its contribution to grain were significantly greater (Table 3). There were no significant differences in DMR between W1 and W3 or W4; however, its contribution to grain was lower in W3 and the differences were significant in 2012–2013. DMA_p_ in W3 was greater than in the W1 and W4, and its contribution to grain was the highest. DMR and its contribution to grain in the W0 were the highest; however, DMA_p_ and its contribution to grain were the lowest in both growing seasons.

**Table 3:** The dry matter remobilization pre-anthesis and accumulation post-anthesis and their contribution to grain of different treatments in 2012–2013 and 2013–2014 growing seasons.

Growing season	Treatment	Dry matter remobilization ^a^ (kg ha^-1^)	CDMRG ^b^ (%)	Post-anthesis dry matter accumulation (kg ha^-1^)	CDMAG ^c^ (%)
2012–2013	W0	2738a	44.0a	3486d	56.0c
	W1	2192c	25.0c	6572b	75.0a
	W2	2381b	33.4b	4747c	66.6b
	W3	2161c	23.3d	7121a	76.7a
	W4	2111c	24.3cd	6592b	75.7a
2013–2014	W0	2161a	37.5a	3596d	62.5c
	W1	1630b	18.6c	7132b	81.4a
	W2	2065a	29.0b	5064c	71.0b
	W3	1728b	18.4c	7668a	81.6a
	W4	1658b	18.9c	7112b	81.1a

^a^ Within a column, values in the same growing season followed by different letters differ significantly (P < 0.05).

^b^ CDMRG: the contribution of dry matter remobilization to grain.

^c^ CDMAG: the contribution of dry matter accumulation post-anthesis to grain.

### Grain filling traits

**Fig 6:**
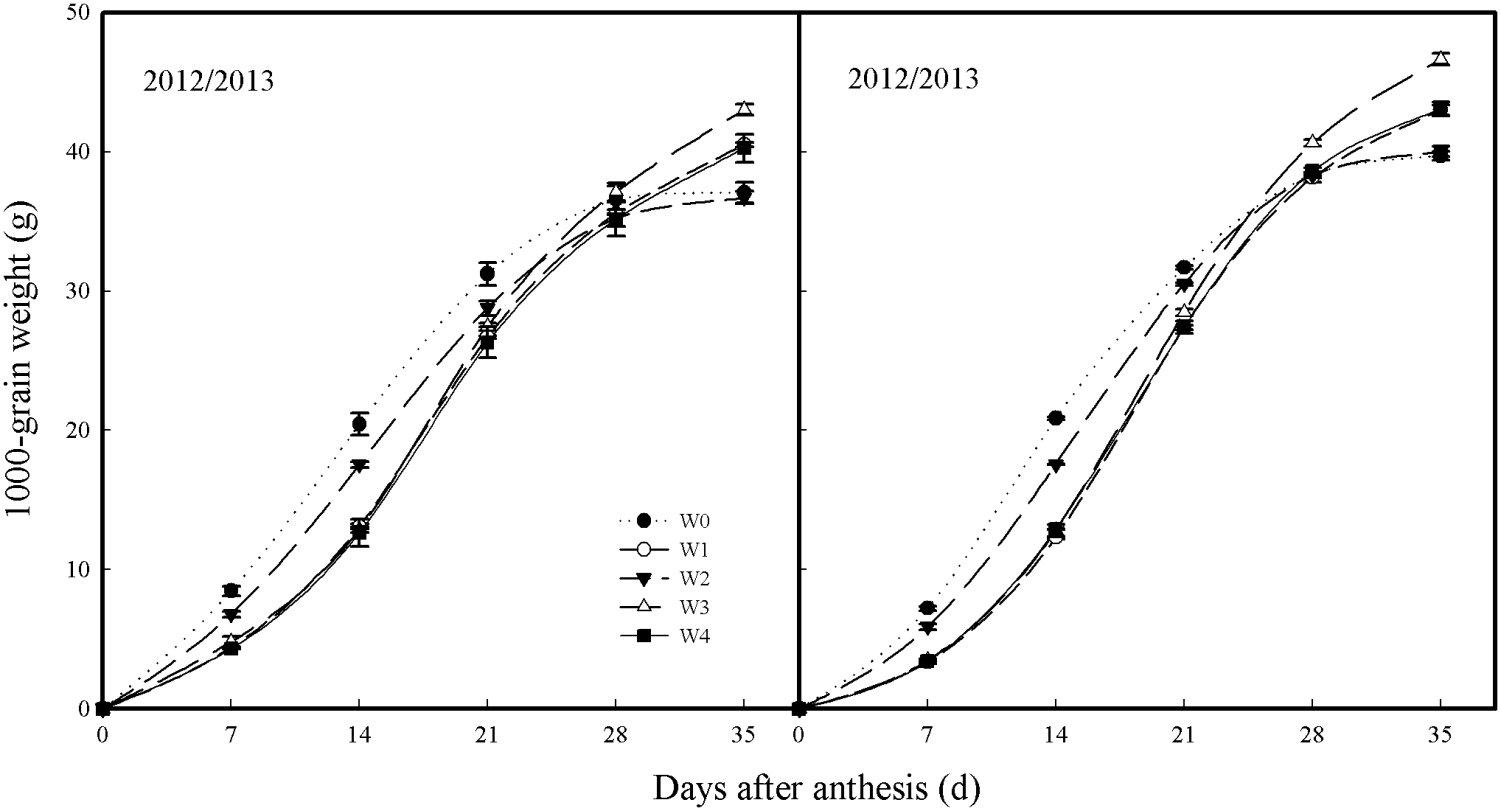
Increase in 1000-grain weight during grain filling in winter wheat plants grown in plots in 2012–2013 and 2013–2014 growing seasons. Error bars represent standard errors of the means (P<0.05).

The accumulation of DM in grain was fitted to a logistic function in the 2012–2013 growing season, which showed similar temporal and spatial variation between and within treatments as that in the 2013–2014 growing season (Fig 6, Table 4). The W3 treatment recorded the highest 1000-grain weight at 28 DAA, followed by the W1 and W4 treatments; the lowest was exhibited by the W0 and W2 treatments. The duration of grain filling (*T*) from the W3 treatment was the highest in both growing seasons; the mean *T* of plants from W3 was greater by 24.2%, 4.3%, 15.9%, and 2.9%, than that of plants from W0, W1, W2, and W4, respectively. In contrast, there was no significant difference in the mean grain filling rate of plants between treatments.

**Table 4:** Logistic equations used to model grain filling and estimates of the duration of grain filling (*T*) and the mean rate of grain filling (Va) for winter wheat plants grown in plots receiving no supplemental irrigation (W0), local SI practice with 60 mm both at jointing and anthesis (W1) or supplemental irrigation determined by measuring 0–20 cm (W2), 0–40 cm (W3) and 0–60 cm (W4) soil profile moisture, respectively.

Growing season	Treatment	Equation	T ^a^ ^,^ ^b^ (day)	Va ^b^ (mg grain^-1^ day^-1^)
2012–2013	W0	GW ^b^ = 37.44/(1+exp(2.92–0.22x))	33.9d	1.10a
	W1	GW = 41.55/(1+exp(3.52–0.19x))	41.1b	1.00a
	W2	GW = 37.17/(1+exp(3.07–0.21x))	36.7c	1.01a
	W3	GW = 44.55/(1+exp(3.52–0.19x))	43.2a	1.03a
	W4	GW = 41.35/(1+exp(3.54–0.19x))	42.0b	0.98a
2013–2014	W0	GW = 39.80/(1+exp(3.07–0.22x))	34.8d	1.14a
	W1	GW = 44.11/(1+exp(3.90–0.21x))	40.7b	1.08a
	W2	GW = 40.63/(1+exp(3.32–0.21x))	36.9c	1.10a
	W3	GW = 48.24/(1+exp(3.87–0.20x))	42.1a	1.15a
	W4	GW = 44.35/(1+exp(3.83–0.21x))	40.9b	1.08a

^a^ Different letters in the same column in the same growing season indicate significant differences at P<0.05.

^b^ GW, 1000-grain weight; T, duration of grain filling; Va, grain filling rate.

### Crop water use and grain yield

The *ETc* of plants from the W1, W3 and W4 did not differ and was significantly higher than that from the W2. The lowest *ETc* was observed in the W0 treatment (Table 5). The 1000-grain weight, grain yield and WUE of plants were highest for the W3, with mean values of 44.7 g, 9169 kg ha^−1^ and 20.8 kg ha^−1^ mm^−1^ in the two growing seasons, respectively. The next highest values were from the W1 and W4, which were significantly higher than those from the W2. The lowest values were from the W0 treatment.

**Table 5:** The crop evapotranspiration (ET_c_), 1000-grain weight, grain yield, and water use efficiency (WUE) in 2012–2013 and 2013–2014 growing seasons.

Growing season	Treatment	ET_c_ ^a^ (mm)	1000-grain weight (g)	Grain yield (kg ha^-1^)	WUE (kg ha^-1^ mm^-1^)
2012–2013	W0	352.1c	37.1c	6123d	17.4d
	W1	432.1a	40.7b	8779b	20.3a
	W2	398.4b	37.7c	7179c	18.0c
	W3	439.4a	42.9a	9077a	20.7a
	W4	442.6a	40.3b	8701b	19.7b
2013–2014	W0	356.0c	40.1c	6407d	18.0e
	W1	445.9a	43.7b	8952b	20.1b
	W2	395.7b	40.2c	7367c	18.6d
	W3	443.4a	46.6a	9260a	20.9a
	W4	453.3a	43.9b	8855b	19.5c

^a^ Within a column, values in the same growing season followed by different letters differ significantly (P < 0.05).

### Correlation analyses

There were significant positive relationships between grain yield, RLD, *P*_n_, *T*_r_, *g*_s_, DMA_p_, GFR and DGF; however, the DMR showed a significant, negative correlation with grain yield, RLD, *P*_n_, *T*_r_, *g*_s_, DMA_p_, GFR and DGF (Table 6). The correlation coefficient between RLD and grain yield was lower than that between DMA_p_ and grain yield and between *P*_n_ and grain yield, but higher than that between the other parameters and grain yield; the DMA_p_ was more highly correlated to grain yield than DMR, the *P*_n_ was more highly correlated to grain yield than *T*_r_ and *g*_s_ and the DGF was more highly correlated to grain yield than GFR.

**Table 6:** The correlation coefficient between grain yield, net photosynthesis rate (*P*_n_), transpiration rate (*T*_r_), stomatal conductance (g_s_), dry matter remobilization (DMR), dry matter accumulation postanthesis (DMA_p_), grain filling rate (GFR), and duration of grain filling (DGF) (n = 30).

	RLD	*P*_n_	*T*_r_	g_s_	DMR	DMA_p_	GFR	DGF
Grain yield	0.894**	0.918**	0.596**	0.698**	-0.645**	0.955**	0.830**	0.888**
RLD		0.881**	0.592**	0.694**	-0.643**	0.892**	0.746**	0.884**
Pn			0.685**	0.785**	-0.495*	0.928**	0.878**	0.894**
Tr				0.596**	-0.337*	0.596**	0.552**	0.686**
Gs					-0.648**	0.892**	0.752**	0.785**
DMR						-0.820**	-0.613**	-0.707**
DMA_p_							0.776**	0.893**
GFR								0.810**

*Significance at P level of 0.05.

**Significance at P level of 0.01

## Discussion

Irrigation significantly affects the soil water conditions. Moderate irrigation can increase RLD, particularly facilitating root growth in the deep soil layers, improving water uptake from soil by crops and increasing the grain yield and WUE of winter wheat, maize, cotton, etc [38–42]. Our previous study by Guo et al. [28] found that the soil water consumption amount of D40 was higher than that of D20 and D60. In the present study, we obtained the same results and found that RLD in the 40–100 cm soil layers at anthesis in W3 was higher than that in the W1, W2 and W4 (Fig 3). It may have been that suitable irrigation at jointing for W3 promoted root growth under 60 cm [43], and this is likely the reason for the higher water consumption in the soil layers under 60 cm in W3; our results were similar to those from a study by Li et al. [14].

Photosynthesis plays a pivotal role in grain yield, and nearly 70% of grain is derived from current photosynthesis in the leaves [44]. An enhanced grain yield of wheat is consistently associated with changes in photosynthetic characteristics, such as *P*_n_, *T*_r_ and *g*_s_ of the flag leaf [45]. The magnitude of the increases in *P*_n_, *T*_r_ and *g*_s_ found in both our previous study by Guo et al. [20] and the present study was similar to reported results concerning winter wheat from studies by Khamssi et al. [46], Sun et al. [47] and Zhang et al. [48], reflecting the beneficial effects of SI in these photosynthetic parameters. Some studies have reported that enhancing *P*_n_ and *g*_s_, particularly at the mid and later stages of grain filling, has significant effects on improving grain yield of crops, such as winter wheat, maize, rice [19, 49–51]. In the present study, we found that the *P*_n_, *T*_r_ and *g*_s_ of flag leaves at 14 DAA from the W3 were significantly higher than those from the W2 and W4, which is similar to the results from the study by Guo et al. [20]. In the present study, we also found that the *P*_n_, *T*_r_ and *g*_s_ of flag leaves for 21–35 DAA from the W3 were significantly higher than those from W1, W2 and W4, which may be beneficial for the high leaf area index at anthesis [20] and high RLD in 40–100 cm soil layers, which promotes soil water absorption (Fig 2 and Fig 3) from the deep soil layers during the grain-filling stages; our findings are similar to those of Hayashi et al. [52]. Therefore, maintaining the soil moisture in the 0–40 cm soil profile at 65% FC at jointing and 70% FC at anthesis facilitated root growth, which provided moderate soil moisture for flag leaf photosynthesis, particularly at the mid and later stages of grain filling, which is conducive to increasing grain weight.

Liu et al. [53] and Ercoli et al. [23] observed that suitable irrigation increased the DMA_p_ and improved the contribution of post-anthesis DMA to grain. Increasing DMA at maturity and improving the contribution of post-anthesis DM to grain can increase the grain yield [17]. We obtained similar results in the present study and further found that the DMA_p_ was more highly correlated to grain yield than DMR. Higher photosynthesis rate post-anthesis, especially at the mid and later grain filling stages, which promoted post-anthesis dry matter accumulation and grain filling (Fig 6), and finally increased the grain weight are likely the reason of high correlation coefficient between DMA_p_ and grain yield. This result was similar to the study on maize by Ning et al. [54]. The DMA post-anthesis, the contribution of post-anthesis DM to grain and DMA at maturity from W3 were significantly higher than those from W1, W2 and W4; these benefits are possibly because of the suitable soil moisture environment in W3 after irrigation at jointing and anthesis. In our previous study by Guo et al. [20], we found that the GFR of D40 was higher than that of D20 and D60 from 14 DAA to 28 DAA. We obtained the same result in this study and we further found that irrigation under W3 not only improved the mean GFR but also significantly increased the DGF compared with the other treatments, which is likely the reason for the higher grain weight in W3, and these benefits are likely accrue through increased root length density in 40–100 cm soil layers. These results indicate that improving the moisture in the 0–40 cm soil profile to 65% FC at jointing and 70% FC at anthesis promotes DMA_p_ and it’s contribution to grain, which correspondingly extending the DGF and possibly increasing DMA at maturity, grain weight and final grain yield for W3.

## Conclusions

SI determined by measuring the moisture in the 0–20 cm (W2), 0–40 cm (W3), and 0–60 cm (W4) soil profiles can regulate SWC to the targeted level. SI under W3 increased RLD in the 60–100 cm soil layers at anthesis and promoted water uptake in the 100–140 cm soil profile from jointing to anthesis and the 60–140 cm soil profile from anthesis to maturity, leading to increased soil water consumption and *ETc*. The maximal net *P*_n_, *T*_r_ and *g*_s_ of flag leaves during the grain-filling stages, particularly at the mid and later stages, were obtained for W3, which increased DMA post-anthesis and it’s contribution to grain, and correspondingly improving the mean GFR, extending the DGF and increasing the grain yield and WUE. In summary, these benefits appear to accrue through increased soil relative water content in the 0–40 cm soil profiles to 65% FC at jointing and 70% FC at anthesis, which created a suitable soil moisture environment for winter wheat production in the Huang-Huai-Hai Plain of China.

## Supporting Information

S1 File The relevant data in Figs 1–6. (XLS)

## References

[1] National Bureau of Statistics of China (2013) China Statistical Yearbook, 449. China Statistics Press, Beijing, pp. 453.

[2] Liu HJ, Yu LP, Luo Y, Wang XP, Huang GH (2011) Responses of winter wheat (*Triticum aestivum* L.) evapotranspiration and yield to sprinkler irrigation regimes. Agric Water Manage 98: 483–492.

[3] Liu HJ, Kang Y, Yao SM, Sun ZQ, Liu SP, Wang QG (2013) Field evaluation on water productivity of winter wheat under sprinkler or surface irrigation in the North China plain. Irrig Drain 62: 37–49.

[4] Yang N, Wang ZH, Gao YJ, Zhao HB, Li KY, Li FC, et al (2014) Effects of planting soybean in summer fallow on wheat grain yield total N and Zn in grain and available N and Zn in soil on the Loess Plateau of China. Eur J Agron 58: 63–72.

[5] Li H, Zheng L, Lei Y, Li C, Liu Z, Zhang S (2008) Estimation of water consumption and crop water productivity of winter wheat in North China Plain using remote sensing technology. Agric Water Manage 95: 1271–1278.

[6] Sun QP, Kröbel R, Müller T, Römheld V, Cui ZL, Zhang FS, et al (2011) Optimization of yield and water-use of different cropping systems for sustainable groundwater use in North China Plain. Agric Water Manage 98: 808–814.

[7] Zhang X, Chen S, Sun H, Shao L, Wang Y (2011) Changes in evapotranspiration over irrigated winter wheat and maize in North China Plain over three decades. Agric Water Manage 98: 1097–1104.

[8] Zwart SJ, Bastiaanssen WGM (2004) Review of measured crop water productivity values for irrigated wheat, rice, cotton and maize. Agric Water Manage 69: 115–133.

[9] Wang D, Yu ZW, White PJ (2013) The effect of supplemental irrigation after jointing on leaf senescence and grain filling in wheat. Field Crops Res 151: 35–44.

[10] Gao Y, Yang LL, Shen XJ, Li XQ, Sun JS, Duan AW et al (2014) Winter wheat with subsurface drip irrigation (SDI): Crop coefficient, water-use estimates, and effects of SDI on grain yield and water use efficiency. Agric Water Manage 146: 1–10.

[11] Zhang XY, Pei D, Chen SY (2004) Root growth and soil water utilization of winter wheat in the North China Plain. Hydrol Process 18: 2275–2287.

[12] Dong BD, Shi L, Shi CH, Qiao YZ, Liu MY, Zhang ZB (2011) Grain yield and water use efficiency of two types of winter wheat cultivars under different water regimes. Agric Water Manage 99: 103–110.

[13] Fang Q, Ma L, Yu Q, Ahuja LR, Malone RW, Hoogenboom G (2010) Irrigation strategies to improve the water use efficiency of wheat–maize double cropping systems in North China Plain. Agric Water Manage 97: 1165–1174.

[14] Li QQ, Dong BD, Qiao YZ, Liu MY, Zhang JW (2010) Root growth available soil water and water-use efficiency of winter wheat under different irrigation regimes applied at different growth stages in North China. Agric Water Manage 97: 1676–1682.

[15] Lv LH, Wang HJ, Jia XL, Wang ZM (2011) Analysis on water requirement and water-saving amount of wheat and corn in typical regions of the North China Plain. Front Agric China 5: 556–562.

[16] Li Q, Liu M, Zhang J, Dong B, Bai Q (2009) Biomass accumulation and radiation use efficiency of winter wheat under deficit irrigation regimes. Plant Soil Environ 55: 85–91.

[17] Zhang XY, Chen SY, Sun HY, Pei D, Wang YM (2008) Dry matter harvest index grain yield and water use efficiency as affected by water supply in winter wheat. Irrig Sci 27: 1–10.

[18] Zheng CF, Jiang D, Liu FL, Dai TB, Jing Q (2009) Effects of salt and waterlogging stresses and their combination on leaf photosynthesis, chloroplast ATP synthesis, and antioxidant capacity in wheat. Plant Sci 176: 575–582. 26493148 10.1016/j.plantsci.2009.01.015

[19] Xu ZZ, Zhou GS (2011) Responses of photosynthetic capacity to soil moisture gradient in perennial rhizome grass and perennial bunchgrass. BMC Plant Biol 11: 21 doi: 10.1186/1471-2229-11-21 21266062 10.1186/1471-2229-11-21 PMC3037845

[20] Guo Z, Yu Z, Wang D, Shi Y, Zhang Y (2014) Photosynthesis and winter wheat yield responses to supplemental irrigation based on measurement of water content in various soil layers. Field Crops Res 166: 102–111.

[21] Zhao D, Shen J, Lang K, Liu Q, Li Q (2013) Effects of irrigation and wide-precision planting on water use, radiation interception, and grain yield of winter wheat in the North China Plain. Agric Water Manage 118: 87–92.

[22] Yang JC, Zhang JH, Wang ZQ, Zhu QS, Liu LJ (2004) Activities of fructan- and sucrose-metabolizing enzymes in wheat stems subjected to water stress during grain filling. Planta 220: 331–343. 15290295 10.1007/s00425-004-1338-y

[23] Ercoli L, Lulli L, Mariotti M, Masoni A, Arduini I (2008) Post-anthesis dry matter and nitrogen dynamics in durum wheat as affected by nitrogen supply and soil water availability. Eur J Agron 28: 138–147.

[24] Han ZJ, Yu ZW, Wang D, Zhang YL (2010) Effects of supplemental irrigation based on testing soil moisture on dry matter accumulation and distribution and water use efficiency in winter wheat. Acta Agron Sin 36: 457–465.

[25] Zadoks JC, Chang TT, Konzak CF (1974) A decimal code for the growth stages of cereals. Weed Res 6: 415–421.

[26] Jalilian J, Modarres-Sanavy SAM, Saberali SF, Sadat-Asilan K (2012) Effects of the combination of beneficial microbes and nitrogen on sunflower seed yields and seed quality traits under different irrigation regimes. Field Crops Res 127: 26–34.

[27] Gardner WH (1986) Water content In: Klute A (Ed), Methods of Soil Analysis, Part 1 Agronomy Monographs 9, 2nd ed Verlag, American Society of Agronomy and Soil Science Society of America, Madison (Wisconsin), pp: 493–544.

[28] Chattaraj S, Chakraborty D, Garg RN, Singh GP, Gupta VK, Singh S, et al (2013) Hyperspectral remote sensing for growth-stage-specific water use in wheat. Field Crops Res 144: 179–191.

[29] Ali MH, Hoque MR, Hassan AA, Khair A (2007) Effects of deficit irrigation on yield water productivity and economic returns of wheat. Agric Water Manage 92: 151–161.

[30] Xue Q, Zhu Z, Musick JT, Stewart BA, Dusek DA (2003) Root growth and water uptake in winter wheat under deficit irrigation. Plant Soil 257: 151–161. 10.1016/j.jplph.2005.04.026 16399006

[31] Zhang XY, Chen SY, Sun HY, Wang YM, Shao LW (2009) Root size, distribution and soil water depletion as affected by cultivars and environmental factors. Field Crops Res 114: 75–83.

[32] Qi WZ, Liu HH, Liu P, Dong ST, Zhao BQ, So NB, et al (2012) Morphological and physiological characteristics of corn (*Zea mays* L.) roots from cultivars with different yield potentials. Eur J Agron 38: 54–63.

[33] Arduini I, Masoni A, Ercoli L, Mariotti M (2006) Grain yield, and dry matter and nitrogen accumulation and remobilization in durum wheat as affected by variety and seeding rate. Eur J Agron 25: 309–318.

[34] Masoni A, Ercoli L, Mariotti M, Arduini I (2007) Post-anthesis accumulation and remobilization of dry matter nitrogen and phosphorus in durum wheat as affected by soil type. Eur J Agron 26: 179–186.

[35] Meade KA, Cooper M, Beavis WD (2013) Modeling biomass accumulation in maize kernels. Field Crops Res 151: 92–100.

[36] Wu WM, Chen HJ, Li JC, Wei FZ, Wang SJ, Zhou XH (2012) Effects of nitrogen fertilization on chlorophyll fluorescence parameters of flag leaf and grain filling in winter wheat suffered waterlogging at booting stage. Acta Agronomica Sinica 38: 1088–1096.

[37] Wang J, Liu WZ, Dang TH (2011) Responses of soil water balance and precipitation storage efficiency to increased fertilizer application in winter wheat. Plant Soil 347: 41–51.

[38] Wang CY, Liu WX, Li QX, Ma DY, Lu HF, Feng W, et al (2014) Effects of different irrigation and nitrogen regimes on root growth and its correlation with above-ground plant parts in high-yielding wheat under field conditions. Field Crops Res 165: 138–149.

[39] Ram H, Dadhwal V, Vashist KK, Kaur H (2013) Grain yield and water use efficiency of wheat (*Triticum aestivum* L.)in relation to irrigation levels and rice straw mulching in North West India. Agric Water Manage 128: 92–101.

[40] Iqbal MA, Shen Y, Stricevic R, Pei H, Sun H, Amiri E, et al (2014) Evaluation of the FAO AquaCrop model for winter wheat on the North China Plain under deficit irrigation from field experiment to regional yield simulation. Agric Water Manage 135: 61–72.

[41] Li C, Sun J, Li F, Zhou X, Li Z, Qiang X, et al (2011) Response of root morphology and distribution in maize to alternate furrow irrigation. Agric Water Manage 98: 1789–1798.

[42] Ning S, Shi J, Zuo Q, Wang S, Ben-Gal A (2015) Generalization of the root length density distribution of cotton under film mulched drip irrigation. Field Crops Res 177: 125–136.

[43] Lv G, Kang Y, Li L, Wan S (2010) Effect of irrigation methods on root development and profile soil water uptake in winter wheat. Irrig Sci 28: 387–398.

[44] Saeidi M, Moradi F, Jalali-Honarmand S (2012) The effect of post anthesis source limitation treatments on wheat cultivars under water deficit. Aust J Crop Sci 6: 1179–1187.

[45] Dahal K, Knowles Vl, Plaxton WC, Hüner NPA (2014) Enhancement of photosynthetic performance water use efficiency and grain yield during long-term growth under elevated CO_2_ in wheat and rye is growth temperature and cultivar dependent. Environ Exp Bot 106: 207–220.

[46] Khamssi NN, Najaphy A (2012) Comparison of photosynthetic components of wheat genotypes under rain-fed and irrigated conditions. Photochem Photobiol 88: 76–80. doi: 10.1111/j.1751-1097.2011.01008.x 21967636 10.1111/j.1751-1097.2011.01008.x

[47] Sun YY, Wang XL, Wang N, Chen YL, Zhang SQ (2014) Changes in the yield and associated photosynthetic traits of dry-land winter wheat (*Triticum aestivum* L.) from the 1940s to the 2010s in Shaanxi Province of China. Field Crops Res 167: 1–10.

[48] Zhang BB, Liu WZ, Chang SX, Anyia AO (2010) Water-deficit and high temperature affected water use efficiency and arabinoxylan concentration in spring wheat. J Cereal Sci 52: 263–269.

[49] Rajala A, Hakala K, Mäkelä P, Muurinen S, Peltonen-Sainio P (2009) Spring wheat response to timing of water deficit through sink and grain filling capacity. Field Crops Res 114: 263–271.

[50] Shimono H, Hasegawa T, Fujimura S, Iwama K (2004) Responses of leaf photosynthesis and plant water status in rice to low water temperature at different growth stages. Field Crops Res 89: 71–83.

[51] Wang RF, An DG, Xie QE, Jiang GM, Wang KJ (2009) Leaf photosynthesis is enhanced in normal oil maize pollinated by high oil maize hybrids. Ind Crops Prod 29: 182–188.

[52] Hayashi T, Yoshida T, Fujii K, Mitsuya S, Tsuji T, Okada Y, et al (2013) Maintained root length density contributes to the waterlogging tolerance in common wheat (*Triticum aestivum* L.). Field Crops Res 152: 27–35.

[53] Liu LP, Ouyang Z (2012) Effects of irrigation schedules on photosynthetic carbon assimilation of winter wheat (*Triticum aestivum* L.) in North China Plain: from leaf to population. J Northeast Agric Univ 19: 20–29.

[54] Ning P, Li S, Yu P, Zhang Y, Li C (2013) Post-silking accumulation and partitioning of dry matter, nitrogen, phosphorus and potassium in maize varieties differing in leaf longevity. Field Crops Res 144: 19–27.

